# shRNA target prediction informed by comprehensive enquiry (SPICE): a supporting system for high-throughput screening of shRNA library

**DOI:** 10.1186/s13637-016-0039-8

**Published:** 2016-02-19

**Authors:** Kenta Kamatuka, Masahiro Hattori, Tomoyasu Sugiyama

**Affiliations:** 1 Graduate School of Bionics, Tokyo University of Technology, 1401-1 Katakura-machi, Hachioji, Tokyo 192-0982 Japan; 2 RNAi Inc., 4-1-4 Hongo, Bunkyo-ku, Tokyo 113-0033 Japan

## Abstract

RNA interference (RNAi) screening is extensively used in the field of reverse genetics. RNAi libraries constructed using random oligonucleotides have made this technology affordable. However, the new methodology requires exploration of the RNAi target gene information after screening because the RNAi library includes non-natural sequences that are not found in genes. Here, we developed a web-based tool to support RNAi screening. The system performs short hairpin RNA (shRNA) target prediction that is informed by comprehensive enquiry (SPICE). SPICE automates several tasks that are laborious but indispensable to evaluate the shRNAs obtained by RNAi screening. SPICE has four main functions: (i) sequence identification of shRNA in the input sequence (the sequence might be obtained by sequencing clones in the RNAi library), (ii) searching the target genes in the database, (iii) demonstrating biological information obtained from the database, and (iv) preparation of search result files that can be utilized in a local personal computer (PC). Using this system, we demonstrated that genes targeted by random oligonucleotide-derived shRNAs were not different from those targeted by organism-specific shRNA. The system facilitates RNAi screening, which requires sequence analysis after screening. The SPICE web application is available at http://www.spice.sugysun.org/.

**Electronic supplementary material:**

The online version of this article (doi:10.1186/s13637-016-0039-8) contains supplementary material, which is available to authorized users.

## Introduction

Reverse genetics approaches, which enable the determination of gene function by analyzing loss-of-function in a phenotype, have been useful for investigating the role of genes in cells and organisms [1, 2]. Recent progress in whole genome sequencing and comprehensive expressed complementary DNA (cDNA) sequencing has enabled the use of systematic approaches to uncover the roles of genes that have been categorized as unknown function genes. Reverse genetics approaches, such as gene knockout with homologous recombination and gene knockdown with antisense RNA, have been highly effective; however, they do not yield rapid results as gene silencing using double-stranded RNA (dsRNA) under RNA interference (RNAi). RNAi allows obtaining loss-of-function phenotypes with high efficiency and specificity within a short period in a wide range of organisms.

Genome-wide reverse genetics performed by RNAi was first demonstrated in *Caenorhabditis elegans* to investigate genes involved in development [1]. Remarkably, the method required no laborious processes to obtain efficient induction of RNAi in the organism. For example, RNAi induction was demonstrated by feeding animals with *Escherichia coli* expressing dsRNA. The strategy also worked in other organisms such as planaria, a model animal for regeneration [3, 4]. However, the use of cDNA-derived dsRNA has been limited to invertebrates because long dsRNAs (>30 nucleotides (nt)) evoke interferon responses in vertebrates. This problem was solved by using small interfering RNA (siRNA) comprising ~21 nt, i.e., 19 bp with 2-nt 3′ overhangs [5]. siRNAs could also be transformed from short hairpin RNA (shRNA) within transfected cells. The findings led to the development of siRNA-directed reverse genetics methods, which included RNAi library construction and screening systems [2, 6–8]. Methodological progress has also revealed that the efficiency of knockdown depends on sequence within each siRNA [9, 10]. Consequently, algorithms were developed to find efficient sequences from genome databases for RNAi and were utilized to design synthetic siRNA oligonucleotides. Associated web applications using these algorithms have facilitated the analysis of loss-of-function phenotypes [11–13].

Because RNAi elicits a sequence-specific knockdown of gene expression, it is reasonable to associate the phenotype observed following siRNA-mediated knockdown with the biological functions of the target gene. Thus, most RNAi libraries were constructed using natural sequences specific to a known gene [14, 15] based on the original theory that siRNAs would specifically recognize the target mRNA without any mismatch between the target sequence and the guide strand of the siRNA. However, off-target silencing by siRNA occurs similar to that observed during silencing by micro RNA (miRNA) [16], suggesting non-assured specificity of siRNAs in the RNAi library. An siRNA would recognize a specific target gene, while also recognizing sequences of other genes with a few mismatches. Thus, several positions within a target gene might need to be further examined. On the other hand, some libraries were constructed using random oligonucleotides harboring artificial sequences that might include both specific and non-specific siRNAs to known genes [7, 17–20]. The main feature of these libraries is that every obtained shRNA needs to be subjected to sequence analysis to identify its target gene. If the siRNA sequence includes mismatches to a probable target gene, it would need further validation using additionally prepared shRNAs specific to the target gene. This is not as efficient as the other library; however, it offers the advantage that the library might have no bottleneck on the diversity of sequences because it was prepared using billions of siRNA sequences up to theoretical 4^*n*^, where *n* is the number of random oligonucleotides. In contrast to the general understanding of off-target silencing by siRNA [16], it is possible that the expression of a target gene with sequence mismatch can be specifically silenced by siRNAs. This might facilitate the use of reverse genetics methodology in genomics because construction of RNAi libraries is easy and inexpensive.

Sequence analysis of an obtained shRNA is an important process in the screening system for RNAi libraries generated from random oligonucleotides. Identification of a target gene might be simple for shRNAs carrying natural sequences. However, the identification might be difficult for siRNAs carrying non-specific sequences. A web application might reduce the laborious analysis of sequence databases. Although several bioinformatics tools are available in the public domain, utilizing each of these tools separately for RNAi screening is not practically efficient. Here, we developed an automated web-based analysis and search tool, shRNA target prediction informed by comprehensive enquiry (SPICE), for investigating biological information about shRNA sequences. By integrating known bioinformatics tools [21–27] and additional processing of data for the efficient evaluation of sequence, SPICE displays target candidate genes with sequence alignment as well as information associated with each gene.

## Web application

**Fig. 1:**
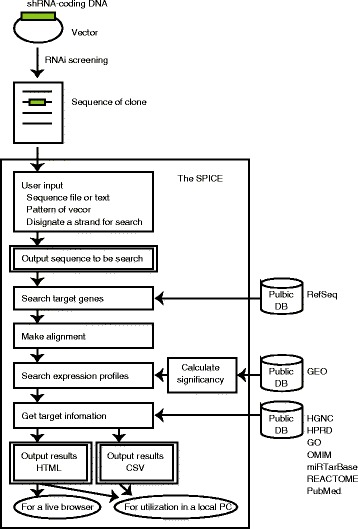
Program flow of SPICE. A sequence obtained from an shRNA-coding DNA clone is processed to extract shRNA sequence. The sequence is subjected to search targets against public database. Biological information on the targets is retrieved from a different database. Result files are generated for use in a local PC

Our goal was to create a web application and provide a website that can support RNAi screening systems using random oligonucleotide RNAi libraries. To this end, the SPICE web application executes several tasks (Fig. 1): (i) identification of siRNA sequence region in vector harboring shRNA-encoding DNA, (ii) sequence alignment between passenger strand of the siRNA and human RefSeq DNA database, (iii) functional annotation of the siRNA target DNA using databases, (iv) calculation of Gene Expression Omnibus (GEO) profile data to show significant microarray experiments in humans, and (v) preparation of downloadable summary files to support spreadsheet database construction in a local personal computer (PC). SPICE can mainly be utilized in RNAi screening using RNAi library that includes random oligonucleotide artificial sequences. It can also be used to investigate possible off-target candidates if the shRNA has a sequence specific to a gene. Case studies as examples of how to use SPICE are included in Additional file 1.

## User input

**Fig. 2:**
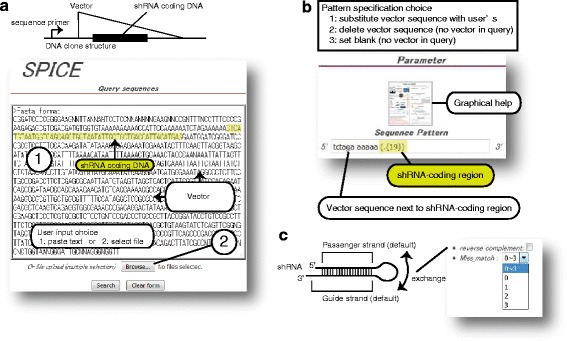
User input of sequence. **a** Uploading FASTA format sequence either from a text box or from a file select button. **b** Search parameter on sequence pattern. **c** Search parameter on strand (passenger or guide) and alignment specificity

In the first step, SPICE accepts either file upload or direct input by replacing the sample sequence that appears as default in a sequence box. For example, after obtaining an shRNA-coding DNA sequence from a sequencer, such as 3130*xl* Genetic Analyzer (Life Technologies/Applied Biosystems, Foster City, CA, USA), the deduced sequence in a FASTA file can be uploaded to the server using a file select button “Query sequences” (Fig. 2a). Alternatively, the FASTA sequence can be copied and pasted to the sequence box. The sequencing direction of forward and reverse in the input sequence does not matter if the sequence is not modified by any other processing because shRNA-encoding DNA consists of inverted repeats [7]. Although the system supports vector sequence harboring shRNA-coding DNA, it should be noted that siRNA only sequence is acceptable by setting “Sequence parameter” either to “blank,” by which the whole input sequence will be used as query, or to “exactly **(.{ 19 })**,” by which the first 19 nt of input sequence will be queried (Fig. 2b). This is a pattern expression of input. The dot in the pattern means any character such as A, T, G, and C except new line. The number between curly braces after the dot specifies the number of occurrences of the dot in the string. The parentheses group characters that were specified by the dot and curly braces. Thus, it is required to set “Sequence parameter” for the siRNA sequence within the input sequence by specifying vector sequence next to the siRNA sequence in the second step. For example, a default sample sequence **tatagaaaaaa(.{ 19 })** shows that an identical vector sequence tatagaaaaaa is followed by a 19-base sequence of siRNA. The sample sequence tatagaaaaaa might be replaced to other vector sequences of sufficient length. SPICE searches vector sequences and identifies an shRNA sequence in the input sequence. In the third step, additional options of “reverse complement” and “Miss_match” can be specified (Fig. 2c). On checking “reverse complement,” the sequence will be queried as a guide strand of siRNA. Although the 5′ portion of shRNA might be a passenger strand in most cases [28], the probability is not 100 % [29]. Therefore, we made it possible to select whether the strand is a passenger or a guide. The “Miss_match” option offers four kinds of search conditions, allowing indicated number of mismatches in alignment between query sequence and that in the database. The default “0–3” mismatch is searched in ascending order until hitting an alignment.

## Identification of possible target genes by primary alignment of siRNA sequence and sequences in database

**Fig. 3:**
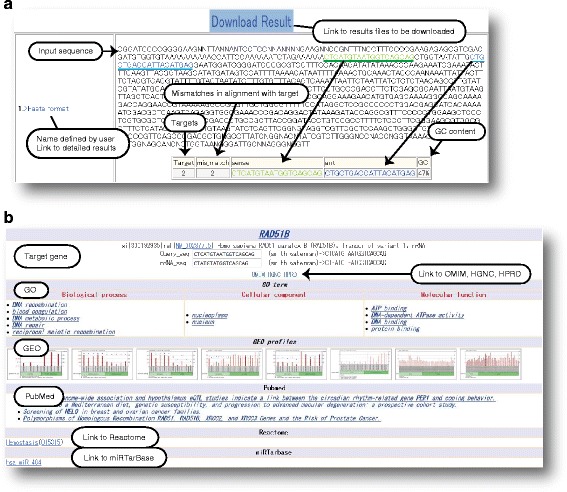
Search result display. **a** Primary information on query shRNA sequence and the URL for the search results. **b** Summary of biological information on shRNA target genes

After execution, by clicking the search button, siRNA guide (antisense) and passenger (sense) strands are extracted from the sequence input. The strands are highlighted in the input sequence and listed with a number of target genes and mismatches, GC content, and a link (sequence name defined in “Query sequences”) to the detailed information window on the target genes (Fig. 3a). The information can be downloaded through “Download Result” for use in a local PC, as described in Fig. 3.

## Prediction of siRNA targets

SPICE predicts target genes by performing GGGenome searches of siRNA sequences against sequences in the human RefSeq database using a parameter of mismatch [27]. GGGenome is an ultrafast search engine for nucleotide sequences and uses the Sedue software (Preferred Infrastructure, Japan) which is useful in handling short sequences. We limited human sequences to experimentally confirmed ones by using records both prefixed with “NM_” and organisms “Homo sapiens.” SPICE selects and shows plus strands from the GGGenome search results because an input siRNA sequence is supposed to be a passenger strand (Fig. 3b). Next, alignment between the siRNA sequence and the selected strand is performed using the algorithm described by Smith and Waterman [30].

## Displaying significant gene expression profiles

To display the expression of profiles of the predicted target genes, SPICE analyzes 361 kinds of selected DataSets of the GEO database [31]. Briefly, GEO contained 1335 kinds of human DataSets among 3413 kinds of whole DataSets. Then, 660 kinds of DataSets were extracted from human DataSets by searching descriptions that compared two experimental conditions with one experimental variable, which was indicated in the subset_type descriptions. Next, 361 kinds of DataSets were chosen as DataSets having more than two samples in each condition. Marked differences between conditions in the expression of each gene in the selected GEO DataSets was previously evaluated using Welch’s *t* test (*P* < 0.01). Therefore, the GEO profiles displayed in the box may exclusively list the novel expression of some subsets under the reported condition (Fig. 3b). Each cartoon of the GEO profiles has a URL for the original source data.

## Links to other databases on siRNA targets

To obtain biological information about the siRNA targets, the name of the siRNA target was searched in each of the following databases: HUGO Gene Nomenclature Committee (HGNC) [32], Human Protein Reference Database (HPRD) [25], Gene Ontology (GO) [21], Online Mendelian Inheritance in Men (OMIM) [23], PubMed, miRTarBase [33], and REACTOME [34]. Links to these databases for each target are provided if there is any relation between the sequence in the database and the siRNA target (Fig. 3b).

## Retrieval of search result files for use in a local PC

**Fig. 4:**
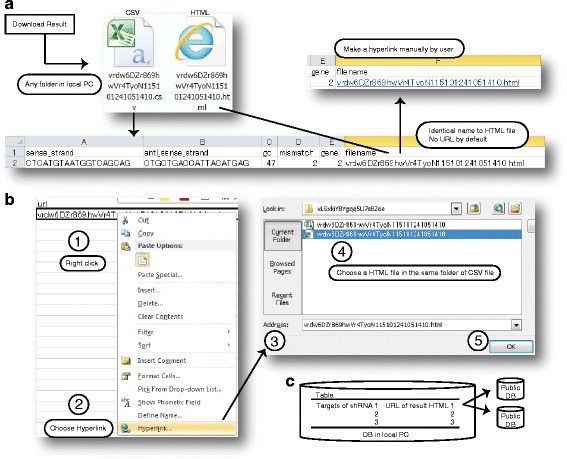
Search result files for utilization in a local PC. **a** CSV and HTML files retrieved from a local PC. **b** Procedure to refer a result HTML file with the name in CSV file. **c** A model of personal instant DB in a local PC

SPICE generates a downloadable compressed file (zip) that includes an HTML file showing the result and a comma-separated value (CSV) table summarizing the siRNA profiles, e.g., sequence, GC contents, number of mismatches with the siRNA, and the name of the HTML file (Fig. 4). These files allow users to retain and utilize the search results in any directory/folder of a local PC. To prevent name redundancy of HTML files, the file is named by assembling 20 randomly chosen characters out of 62 different alphabets and numbers along with the time stamp. The HTML file will show results in any browser without searching again. Because text links to databases are active, original descriptions in the database can be referred. Remarkably, the resultant spreadsheet can be used as a front page of siRNA information by manually hyperlinking an HTML file name in the sheet onto a corresponding HTML file placed in the same folder. This can be easily accomplished using a basic function in a spreadsheet software package. For example, a hyperlink can be made using MS-EXCEL (Microsoft, CA, USA) as follows. (1) Locate the HTML file name in a table. (2) Choose a command “hyperlink…” from the “Insert” tab. (3) Choose the HTML file in the hyperlink insertion pop-up window. (4) Save the EXCEL file. The resultant table should have the HTML file name with the URL. This also facilitates the building of an instant database by combining multiple tables in a single file. Additionally, other comments on siRNA can be included by making new columns in the table.

## Evaluation of a web application

The number of siRNA target candidates was compared between SPICE and other sequence search engines. A representative result for the shRNA GAUUAUCCAAAGAGGUUCU (passenger strand) targeting *RPS6KA6* gene [2] was used. SPICE showed only one target when executed without a mismatch. GGGenome search yielded five candidates including the target with no mismatch. The rest of the candidates were predicted genes that were indexed with “XM_”, which indicate the sequence was predicted as gene by RefSeq. BLAST search showed 100 candidates including the target. Five genes had no mismatch in alignment. The rest of the candidates included one to six mismatches in alignment. Similar results were obtained for another shRNA UGGUUGAUGAGCCAAUGGA (passenger strand) targeting *RPS6KA6* gene [2]. Thus, all of the above applications listed the siRNA target. Of note, SPICE is sufficient for target prediction. Next, we investigated the specificity of the target prediction by using experimentally validated shRNA sequences (Table 1). SPICE showed the identical single target for each sequence, suggesting high specificity of target prediction.

**Table 1:** Specificity of siRNA target prediction

shRNA (passenger)	Target	Reference	Target predicted by the SPICE^a^
GAUUAUCCAAAGAGGUUCU	*RPS6KA6*	Berns et al. [2]	*RPS6KA6* (1, 0)
UGGUUGAUGAGCCAAUGGA	*RPS6KA6*	Berns et al. [2]	*RPS6KA6* (1, 0)
GUACGGCCGUAGUCUCAAG	*HTATIP*	Berns et al. [2]	*KAT5* (1, 0): identical to *HTATIP*
CAAACGUCUGGAUGAAUGG	*HTATIP*	Berns et al. [2]	*KAT5* (1, 0): identical to *HTATIP*
GCCAUCCAGAUGGACUUUC	*HDAC4*	Berns et al. [2]	*HDAC4* (1, 0)
GCAUGUGUUUCUGCCUUGC	*HDAC4*	Berns et al. [2]	*HDAC4* (1, 0)
GCAGCAGAGGUGAUUCUGC	*CCT2*	Berns et al. [2]	*CCT2* (1, 0)
GAGAGGCGCUGUUGAGUUC	*CCT2*	Berns et al. [2]	*CCT2* (1, 0)
GAGUACAUUCUGCCUUGCU	*KIAA0828*	Berns et al. [2]	*AHCYL2* (1, 0): identical to *KIAA0828*
GCUACAUCAAGAACUCAGC	*KIAA0828*	Berns et al. [2]	*AHCYL2* (1, 0): identical to *KIAA0828*
GAGACCTACCTCCGGATCA	*PLK1*	Jackson et al. [16]	*PLK1* (1, 0)

^a^Numbers in parentheses show (target genes, mismatches in alignment)

Next, we investigated gene expression profiles obtained using GEO. For example, there were 5245 kinds of GEO profiles on *RPS6KA6* gene in the current GEO database. The number was decreased by 3577 using a filter “Organism human.” Additionally, the number was decreased by 42 using a filter “Differential expression Up/down genes.” On the other hand, SPICE displayed 16 profiles that were confirmed manually using the original values in GEO profile data. We found that there was no overwrap between the results, suggesting different sensitivities for the selections. Not surprisingly, the differential expression profile shown by SPICE might be only a part of the complete expression profile of the targeted gene.

Estimated time for receiving search results was 6 to 12 s per siRNA target gene. The time depends on how many targets an siRNA sequence has in the database. Because SPICE first searches targets with no mismatch and continues the process with mismatches until it finds a target, the number of target genes increased when searching with mismatches. It took approximately 10.5 min to search 76 target genes for an siRNA sequence.

## Evaluation of random shRNA library using a web application

SPICE was developed for searching targets of shRNA obtained using random oligonucleotides. However, the characteristics of the shRNA sequence were not analyzed thoroughly. It is not clear how many shRNA clones from the RNAi library are sufficient to investigate all human genes. Therefore, we analyzed 47 clones obtained from an RNAi library constructed using random oligonucleotides (Table 2). Each sequence shows the DNA encoding the passenger strand of the shRNA. Interestingly, 19-nt sequences showed no perfect alignment with sequences in human RefSeq database (Table 2). By allowing a mismatch in the alignment, target genes increased from zero to four. Most of the sequences needed two to three mismatches to find targets. These results suggested that to obtain perfectly matched shRNA to any gene during RNAi screening, 47 times the number of shRNA clones against human genes might not be sufficient to cover all human genes.

**Table 2:** Number of target candidate genes against shRNA constructed using random oligonucleotides

	Number of aligned genes with mismatches			
Sequences of shRNA-coding DNA	0^a^	1^a^	2^a^	3^a^
GGTAGCTCGTTAATTTAAT	0	0	1	3
GAATTCCGCGGTACGGGGG	0	0	0	6
GAATGCAGCAGGAGGGAGG	0	1	8	278
GAACTGGACGCAGGCCTAT	0	0	2	53
GACTAGTCTACTTCCTTAG	0	0	0	31
GCGATCTCTACCACCTGGC	0	0	4	79
GCGTAATCGGGCGAATAAC	0	0	0	0
GATAGTGCACGTTGAAACA	0	0	1	38
GTAGGGGGACCTAGAACAT	0	0	2	20
GACAAACCCTGCCTAGTTA	0	0	0	17
GGATCCGAGTGCACCTAAA	0	0	0	28
GAAGTAATGCGCTCGATCC	0	0	1	13
GCCACGTATGGCCGTACTG	0	0	0	10
GCACAATGTAAAAAGAATA	0	2	5	131
GAGCCAGTAAAAGAATATC	0	1	4	55
GTGCACTACTCGCAGTGGG	0	0	2	34
GTTCCTAGACTTCAAATAA	0	0	2	53
GACCTACCCATGTAGCAAG	0	0	0	43
GAGAAGGCGTCTCCGGAGA	0	0	4	82
GAGGAATGATATGGCCTTG	0	0	1	54
GGGGCAGTGGCAGGTGATC	0	1	7	140
GACCAGGGACACATTAGAT	0	0	1	37
GATCTAAATATGGCAGGCA	0	0	0	44
GAAGACAGACACGGAGATC	0	0	7	112
GTCAGCCAGCAAAGAAGTG	0	0	7	115
GTGGACTAGTATCGATATA	0	0	1	10
GTGAACCAAGTTACTAGCT	0	0	0	39
GGCCCGGTTAGGAACACTT	0	0	0	7
GGCCAAGAAGATACACTAG	0	0	1	65
GGACAGGGCAACAATGTGG	0	0	1	44
GGAAGATATGCTCATTCGC	0	0	0	24
GGAAGTGGTAAGGACTCCA	0	0	3	65
GGAGTCAGTACAGATACGA	0	0	1	22
GCGTTAATTAATCAACATC	0	0	0	12
GGCGTGATCGTATGCACCA	0	0	0	13
GAGAAGTCCAGATCTTTCC	0	0	5	95
GGACCCTGTCCCTAGAAGT	0	0	2	72
GAAAACTGTCAGACCCGAG	0	0	1	46
GCATTCCAAAGAAAAGAAT	0	0	7	167
GTCACATATCCTGGACACG	0	0	1	36
GTGTATAGTGCAAGACAAT	0	0	0	48
GTGAGAACGTTGGAGAGCC	0	0	1	59
GTTTTGATCGGAGAATCCA	0	0	1	31
GCAAACATCCAGAAAGTAA	0	0	1	93
GTCCAGGGGTTGGGGATCG	0	0	1	34
GGGCGTAGGTCAAAGCATG	0	0	0	17
GGGCGGGGTAAAGAAACCA	0	0	0	17

Alignment between shRNA and human RefSeq was performed under conditions in which the indicated number of mismatches was allowed

^a^Represents number of mismatched nucleotides

**Fig. 5:**
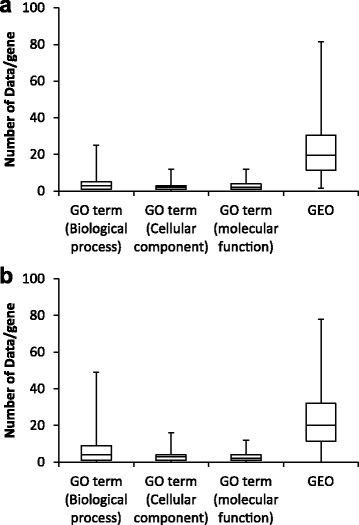
Profiles of genes targeted by organism-specific shRNAs and by shRNAs showing partial specificity. Number of GO and GEO associated with target genes was calculated to compare gene profiles. **a** Human RefSeq genes that were randomly selected as organism-specific shRNA targets. *n* = 139. **b** Human RefSeq genes for each gene were aligned to shRNA with one or two mismatches. *n* = 67

Because most shRNA sequences in an RNAi library constructed using random oligonucleotides are not specific to the sequences in an organism, as described above, it is not assured that these shRNAs would target a series of genes as organism-specific shRNAs would. To investigate the similarity, we compared the profiles of genes targeted by organism-specific shRNAs with those of genes targeted by shRNAs derived from the RNAi library. We used 139 randomly selected human RefSeq sequences as representative targets of organism-specific shRNA. shRNAs from the RNAi library (Table 2) were used as non-organism-specific shRNA. Genes in the human RefSeq database included one to two GO terms (median) (Fig. 5a). Approximately 18 GEO profiles were associated with a gene whose expression was significantly different among subsets. Comparably, shRNA targets shown in Table 2 showed a similar distribution (Fig. 5b). Thus, targets of shRNAs randomly derived from an RNAi library are not different from those of organism-specific shRNAs.

## Conclusions

We have developed SPICE and provided the website for supporting RNAi screening systems using random oligonucleotide RNAi libraries. The SPICE web application can show siRNA target DNA with sequence alignment and the functional annotation. It also provides the downloadable summary files for database construction in local PC. SPICE can be used to facilitate sequence analysis of siRNAs carrying non-specific sequences to natural sequences that will be obtained in RNAi screening.

## Additional file

Additional file 1:
**Search examples.** Execution using siRNA sequence or a sequence file form sequencer. (DOCX 464 kb)
