# Supplementary material for: shRNA target prediction informed by comprehensive enquiry (SPICE): a supporting system for high-throughput screening of shRNA library
Source: EURASIP J Bioinform Syst Biol. 2016 Feb 19;2016:7. doi: 10.1186/s13637-016-0039-8 (PMC4761003; doi:10.1186/s13637-016-0039-8)
Supplement: Additional file 1: — Search examples. Execution using siRNA sequence or a sequence file form sequencer. (DOCX 464 kb) [file 13637_2016_39_MOESM1_ESM.docx]

**SEARCH EXAMPLE 1**

Query sequence: ACCATGTAATGGTAGCAGT (passenger strand of a siRNA)

Name of sequence: siRNA1

(1) Input sequence from a text box


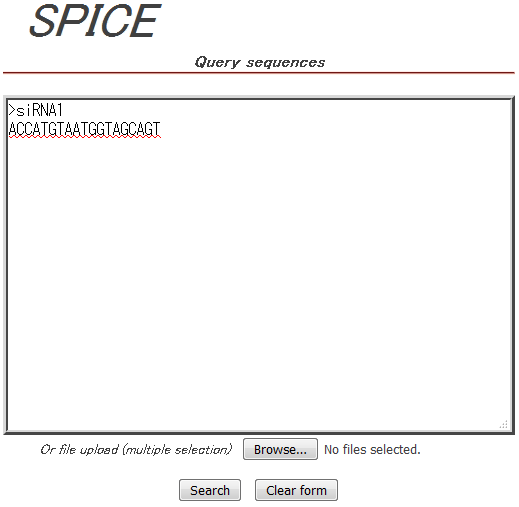


(2) Set parameters

**Pattern**: blank

**Reverse complement**: no check (meaning use of the sequence as a passenger)

**Miss_match**: select one of four (0-3: sequential testing from 0 to 3, 0: no mismatch, 1: a mismatch, 2 two mismatch, 3: three mismatch)


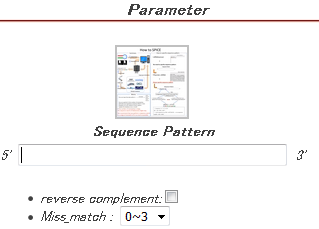


(3) Execute "Search" button.

(4) Display of search results

**Top "Download Result":** a link to result CSV and HTML files for save in local PC

**Left column**: a link to result web page

**Right column**: sequence for searching public database, statics of searched result


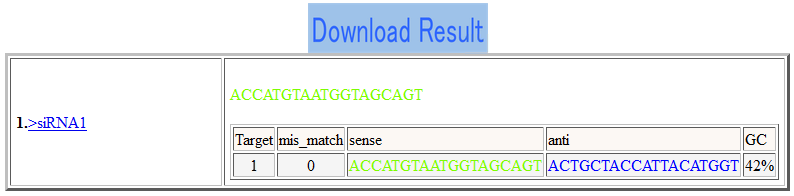


(5) Display of siRNA target genes information


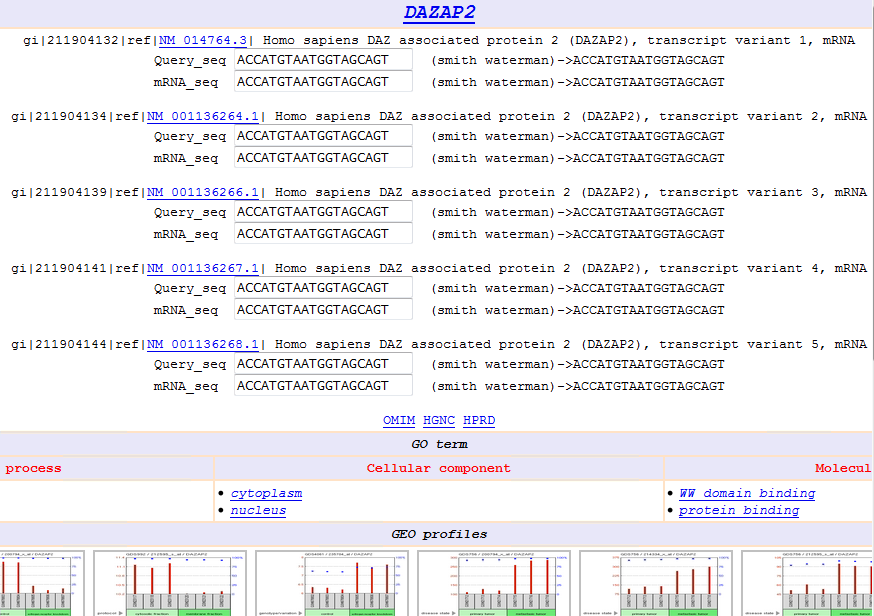


(6) Utilization of CSV and HTML result files in a local PC

Retrieve a compressed file including CSV and HTML files at any folder in a local PC

Expand the compressed file

Open the CSV with MS-EXEL


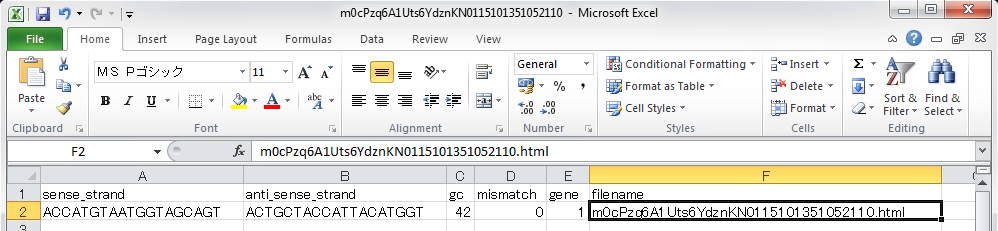


Make a hyperlink from HTML name in the spreadsheet to the HTML file


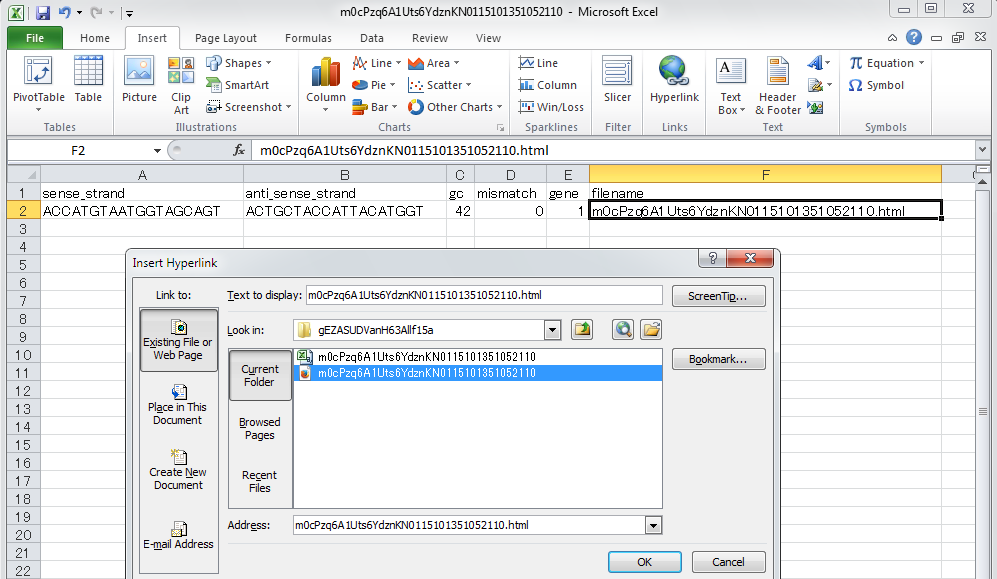


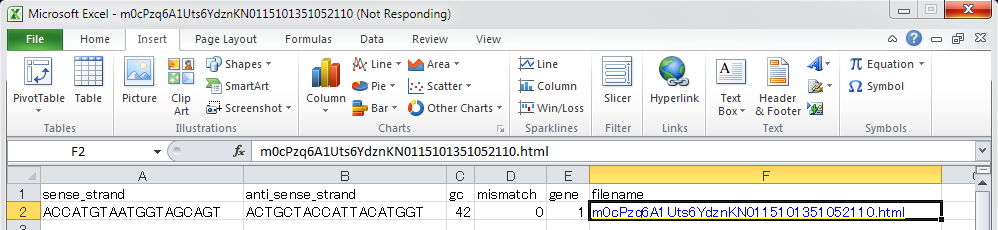


**SEARCH EXAMPLE 2**

Query sequence file: shRNA_clone_1.seq (an outputted sequence file from a sequencer)

(1) Input sequence from "Browse" button

Delete sequence in text box


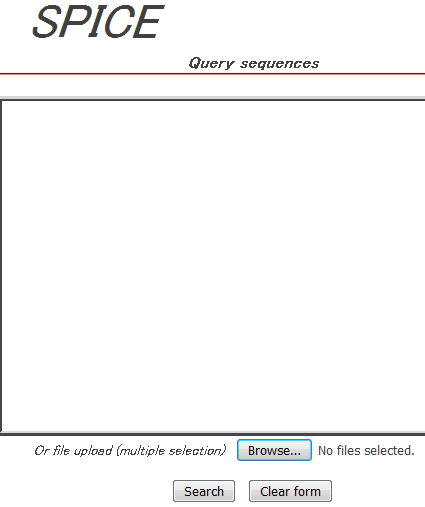

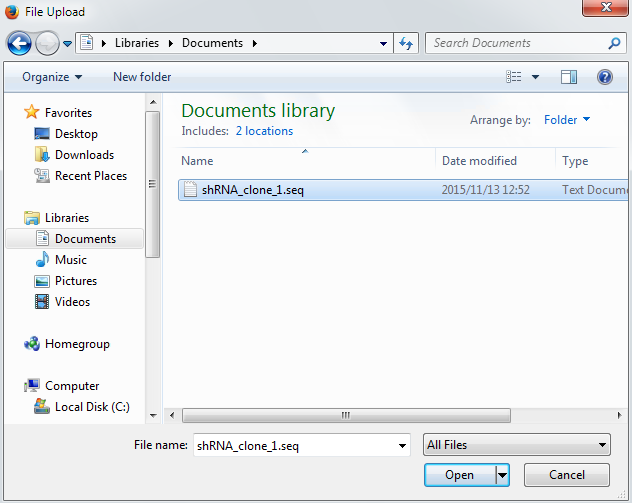


(2) Follow the same process as described in (2) of SEARCH EXAMPLE 1
